# Development of the Online Problem Gaming Behavior Index: A New Scale Based on Actual Problem Gambling Behavior Rather Than the Consequences of it

**DOI:** 10.1177/01632787231179460

**Published:** 2023-05-27

**Authors:** Michael Auer, Neven Ricijas, Valentina Kranzelic, Mark D. Griffiths

**Affiliations:** 1 Neccton GmbH, Lienz, Austria; 2 Faculty of Education and Rehabilitation Sciences, Department of Behavioural Disorders, 37631 University of Zagreb, Campus Borongaj, Zagreb, Croatia; 3 International Gaming Research Unit, Psychology Department, 6122 Nottingham Trent University, Nottingham, UK

**Keywords:** Problem gambling, Online gambling, Gambling screens, Problem Gambling Severity Index, Online Problem Gambling Behavior Index

## Abstract

Many items in current problem gambling screens focus on negative consequences of gambling and gambling-related harms. However, few problem gambling screens comprise items that are totally based on actual gambling behavior such as gambling duration, gambling frequency, or gambling late at night. The aim of the present study was to develop and validate the 12-item Online Problem Gambling Behavior Index (OPGBI). A total of 10,000 online Croatian gamblers were administered the OPGBI alongside the nine-item Problem Gambling Severity Index (PGSI), as well as questions regarding types of gambling engaged in and socio-demographic factors. The 12 OPGBI items mainly concern actual gambling behavior. The correlation between OPGBI and PGSI was highly significant (*r* = 0.68). Three latent factors in the OPGBI were identified (gambling behavior, limit setting, communication with operator). The three factors all significantly correlated with the PGSI score (*R*^2-^ = 51.8%). The fact that pure gambling behavior related items explained over 50% of the PGSI score strengthens the idea that player tracking could be an important approach in identifying problem gambling.

## Introduction

There are now over 30 different instruments that assess disordered gambling (Dowling et al., 2019; Otto et al., 2020). One of the first screens to be developed to assess problematic forms of gambling was the South Oaks Gambling Screen (SOGS; Lesieur & Blume, 1987) and which has been used in many prevalence studies worldwide. However, methodological and psychometric critiques of the SOGS have claimed that it does not accurately assess problematic forms of gambling within general population settings (Stinchfield et al., 2007). For example, when compared to other screening instruments such as the Problem Gambling Severity Index (PGSI) (Ferris & Wynne, 2001), it has been claimed that the SOGS has a tendency to overestimate the prevalence rates of problem gambling within community-based samples (Stinchfield, 2002). Psychometric instruments such as the PGSI were specifically developed for use in epidemiological studies to estimate problem gambling prevalence rates. Numerous studies have shown that the PGSI has good psychometric properties in terms of its reliability and validity (e.g., Lopez-Gonzalez et al., 2018; McCready & Adlaf, 2006; Orford et al., 2010; Stinchfield et al., 2012), and is viewed by some scholars in the gambling studies field as the ‘gold standard’ in countries such as Australia and Canada where it has been used for collecting data regarding the symptoms of problem gambling (McCready & Adlaf, 2006; Neal et al., 2005).

The unidimensional PGSI comprises nine items and was developed to assess a single latent variable (i.e., problem gambling). The rating on each of the nine equally weighted items (scored from 0 to 3) are added together to provide a total score (out of 27) (Neal et al., 2005; Toce-Gerstein et al., 2003; Williams & Volberg, 2014). A large-scale psychometric analysis of the PGSI by Miller et al. (2013) conducted among a sample of 25,000 Canadians supported the unidimensional model and also found that the screening instrument was invariant across age, gender, income level, gambler type, and income level. Lopez-Gonzalez et al. (2018) also reported satisfactory psychometric properties (including construct validity and external validity) of the PGSI among a sample of 659 sports bettors in their Spanish validation study.

Like the SOGS, the PGSI has also been subject to negative critiques. For instance, Currie et al. (2013) reported that the total score on the PGSI has psychometric properties suggesting a more ordinal – as opposed to scalar – interpretation. Moreover, they also reported that there was poor support in relation to the delineation of the PGSI’s intermediate categories. One explanation may be because items in the PGSI are over-represented by symptoms more associated with disordered gambling as opposed to individuals with lesser gambling problems (McCready & Adlaf, 2006). Another study using a large-scale representative British sample reported that among women, the PGSI underestimates the prevalence rate of problem gambling (Orford et al., 2010).

Although there are some negative consequences that are assessed, in assessing gambling-related harm, the PGSI is primarily based on an addiction-based approach, rather than one based on a public health model. In relation to problem gambling, a public health approach considers that the largest component of community gambling-related harm is not from those with the most severe gambling problems, but from the much larger group experiencing far fewer severe gambling-related problems (Shaffer 2003; Shaffer & Korn, 2002; Svetieva & Walker, 2008). The term “prevention paradox” was coined by Rose (1985, 1992) and assumes that most cases of a disease or disorder (in overall absolute numbers) occur among populations with a large number of lower risk individuals, and that relatively few cases occur among smaller higher risk populations. Consequently, Browne et al. (2018) developed the 10-item Short Gambling Screen (SGH) selected from a more comprehensive 72-item harms checklist of specific gambling-related harms. Moreover, research has found large commonalities in the experience of harms reported by gamblers with those around them (Li et al., 2017).

More recently, Jonsson et al. (2017) developed GamTest which assesses five domains of problem gambling (i.e., social consequences, monetary consequences, overspending of money, overspending of time, and emotional negative consequences). They validated GamTest and compared it with the PGSI in a study of 10,402 Nordic online players. The correlation between summed scores for GamTest and the PGSI was high (*r* = 0.81). Several other less frequently used problem gambling screens have been developed including the Brief Biosocial Gambling Screen (Gebauer et al., 2010), the Victorian Gambling Screen (Ben-Tovim et al., 2001), Lie-Bet Questionnaire (Johnson et al., 1997), and the Sydney Laval Universities Gambling Screen (Blaszczynski et al. (2008).

### Behavioral Aspects of Problem Gambling

Most diagnostic screening instruments for problem gambling focus on either the consequences and/or psychological aspects of gambling. For example, only two of the nine PGSI items are concerned with actual gambling behavior (“*Have you needed to gamble with larger amounts of money to get the same excitement?*” and “*Have you gone back to try to win to back the money you’d lost?*”). Moreover, some researchers in the gambling studies field have emphasized the importance of behavioral aspects in the identification of problem gambling – even before the rise in popularity of online gambling (e.g., Auer & Griffiths, 2023; Catania & Griffiths, 2022; Delfabbro et al., 2012).

In the fifth edition of the *Diagnostic and Statistical Manual of Mental Disorders* (DSM-5; American Psychiatric Association, 2013), one of the criteria for gambling disorder (GD) is preoccupation with gambling (which equates to salience). Salience describes a high preoccupation with an activity and could potentially be asked in any self-report screening instrument. Tolerance is also associated with GD and is defined as the need to “*gamble with increasing amounts of money in order to achieve the desired excitement*” (Lee et al., 2020). Tolerance is a key diagnostic criterion for problem gambling (Lesieur, 1988) and is also a specific question in the PGSI (“*Have you needed to gamble with larger amounts of money to get the same excitement?*”). Chasing has been identified as one of the central characteristics of GD (American Psychiatric Association, 2013). In a survey of 10,838 online gamblers, Gainsbury et al. (2014) reported that online casino players had a greater tendency to report chasing losses than poker players. They also found that players who engaged in chasing losses were more likely to hold irrational beliefs about gambling and spend more time and money gambling than those who did not engage in chasing losses.

Using customer email correspondence data from gamblers who had self-excluded from online gambling websites (*n* = 150) compared to gamblers from the same websites who had not self-excluded (*n* = 150), Haefeli et al. (2011) correctly classified three-quarters of problem gambling cases correctly. Correspondence from the self-excluders was more threatening than from non-self-excluders. In another study by Haefeli et al., 2015, the same email correspondence data were analyzed both manually and with an automated computer text program. The analysis was carried out by classifying words in the written text into specific psychological categories (e.g., positive emotion, anger, anxiety, etc.). The findings indicated that words related to anger and time were predictive of future self-exclusion. Words like ‘hence’ and ‘because’ (which were classed as ‘causation’) were negatively associated with future-self-exclusion. The results also demonstrated that automated text analysis had an improved validity and classification rate compared to the manual rating.

Currently, only a few attempts have been made to develop screens which are based on gambling behavior rather than consequences of problem gambling and psychological aspects of problem gambling. Rockloff (2012) developed the Consumption Screen for Problem Gambling (CSPG) which is based on three items. (i) “*How often did you gamble in the past 12 months?*”, (ii) “*How much time did you spend gambling on a typical day in which you gambled in the past 12 months?*”, and (iii) “*How often did you spend more than 2 hours gambling (on a single occasion) in the past 12 months?*” Scores on the CSPG correlated with scores on the PGSI. Problem gamblers had higher scores than non-problem gamblers. Of the 1,398 participants, 14 of them were problem gamblers (1%) and all of them scored 4+ on the CSPG. Rockloff (2012) concluded that the results demonstrated that the CSPG scale is capable of accurately identifying people with severe gambling problems based on their high levels of gambling consumption. However, the actual number of problem gamblers was few, and arguably, the CSPG only assesses gambling consumption, not specifically gambling harm (or problem gambling).

Brosowski et al. (2021) correlated self-reported gambling intensity for 15 game-types with PGSI responses based on three merged cross-sectional Icelandic gambling surveys. Explanatory variables were grouped into the number of game types played, gambling frequency within the type, maximum gambling frequency across all types beyond, usual spending within the type, and maximum usual spending across all types beyond. The study found that offline electronic gaming machines, offline scratch-cards offline, online live betting, and offline poker as well as online poker increased problem gambling mostly through gambling frequency of that particular game-type, whereas all other types of gambling mostly increased problem gambling through the number of different game types played.

Based on data from 19,012 individuals participating in the Canadian Community Health Survey, Currie et al. (2006) found a positive association between gambling-related harm and (i) gambling frequency, and (ii) volume of gambling. They concluded that the optimal limits for low-risk participation were (i) gambling at most two to three times a month, (ii) spending less than 1% of gross family income on gambling activities, and (iii) spending no more than $501–$1000 (Canadian) per year on gambling.

### The Present Study and Rationale for Developing a New Problem Gambling Screen

Specific aspects of gambling behavior (e.g., increased gambling intensity, chasing losses, etc.) and communication-based aspects of gambling (e.g., verbally aggressive email correspondence) have been emphasized as indicators of problem gambling (Catania & Griffiths, 2022; Hopfgartner et al., 2023; Ukhov et al., 2021). The rationale for a new screen is that items in the most used current problem gambling screens primarily focus on consequences of problem gambling and psychological aspects of problem gambling rather than the behavior itself. Moreover, some of the items concentrate on very negative and detrimental consequences which may lead to gamblers providing socially desirable answers (e.g., “*Lies to conceal the extent of involvement with gambling”, “Relies on others to provide money to relieve desperate financial situations caused by gambling”, “Has jeopardized or lost a significant relationship, job, or educational or career opportunity because of gambling”* etc.). Therefore, the goal of the present study was to develop a new problem gambling screen (i.e., the Online Problem Gambling Behavior Index [OPGBI]) comprising items that mostly concerned the gambling behavior itself rather than the psychological or behavioral consequences, with items that lower the likelihood of providing socially desirable answers. It also aimed to include questions that were very specific. For instance, one of the few questions concerning actual gambling behavior in the PGSI (about chasing losses) is *“Have you gone back to try to win to back the money you’d lost?”* This question does not differentiate between players who try to win back their money within-session or those players who come back the next day to win their money back. Another key advantage to developing this new instrument is that all the behaviors (i.e., markers of gambling harm) can be identified by online gambling operators using online account-based tracking data. This means that they too could screen for problem gambling using the new screen’s indicators as markers of harm. In developing a new screening instrument for problem gambling based purely on actual gambling behavior, the main aim was to test the efficacy of the OPGBI in identifying problem gambling.

## Methods

### Participants, procedure, and ethics

The study participants comprised players who gambled at the Croatian online lottery’s’ website (www.lutrija.hr). Every visitor was prompted with a window which asked to participate in an academic study and if they agreed they were directed to the online survey site. The application used detected the users’ browser language. The website languages are Croatian, English, and German (but the overwhelming majority of participants appeared to be Croatian). The average age of the 10,000 participants was 43 years (SD = 13), and 2,540 participants were female (25.4%), and 72 (0.72%) reported ‘other’ for gender. The study was given approval by the research team’s university ethics committees.

### Measures

*Demographic and gambling variables:* Participants were asked their gender, their age in years, and which types of gambling games they had played online or offline in the past month (multiple selections were possible).

PGSI*:* The PGSI was developed by Ferris and Wynne (2001). Table 1 lists the nine PGSI items (e.g., *“Have you bet more than you could really afford to lose?”*). Participants are asked to answer the PGSI questions thinking about the past 12 months. For each of the nine items players have to choose between the categories ‘Never’ (0), ‘Sometimes’ (1), ‘Most of the time’ (2) and ‘Almost always’ (3) and scores range between 0 and 27. Higher scores indicate greater problems with gambling. Cronbach’s alpha in the present study was excellent (.91).

**Table 1.** Distribution of the Responses to the PGSI and OPGBI (*n* = 10,000)

OPGB	Item	Never, %	Some times, %	Most of the Time, %	Almost Always, %
1	Do you reload your wallet during an online gambling session?	47	40	6	7
2	Do you increase your stakes after losing in an online gambling session?	70	26	2	2
3	Do you increase your stakes the following day after you have lost in an online gambling session?	79	19	1	1
4	Do you gamble online for longer than 4 hours a day?	88	10	1	1
5	Do you gamble online with a variety of different stakes?	60	34	4	3
6	Do you play more than five types of online gambling games in a month?	78	16	2	3
7	Do you re-gamble your online winnings straight after you have won?	41	45	7	7
8	Do you use different debit or credit cards to load up your wallet during an online gambling session?	73	19	3	6
9	Do you act aggressively in online gambling chat rooms?	97	2	0	0
10	Do you contact customer services to complain about your online gambling losses?	97	3	0	0
11	Do you hit your (or the website’s) money spending limits (if you have any)?	76	19	2	3
12	Do you hit your (or the website’s) time spending limits (if you have any)?	83	14	2	2
**PGSI**					
1	Have you bet more than you could really afford to lose?	85	13	1	1
2	Have you needed to gamble with larger amounts of money to get the same excitement?	82	15	1	1
3	Have you gone back to try to win to back the money you’d lost?	73	23	2	2
4	Have you borrowed money or sold anything to get money to gamble?	94	5	0	1
5	Have you felt that you might have a problem with gambling?	78	18	2	2
6	Have you felt that gambling has caused you any health problems, including stress or anxiety	88	10	1	1
7	Have people criticized your betting, or told you that you have a gambling problem, whether or not you thought it is true?	85	13	1	1
8	Have you felt your gambling has caused financial problems for you or your household?	91	7	1	1
9	Have you felt guilty about the way you gamble or what happens when you gamble?	74	21	2	3

OPGBI: Table 1 lists the 12 items related to gambling behavior (e.g., *“Do you re-gamble your online winnings straight after you have won?”*). Participants are asked to answer the 12 questions thinking about the past month. For each of the 12 questions players had to choose between the categories ‘Never’ (0), ‘Sometimes’ (1), ‘Most of the time’ (2) and ‘Almost always’ (3) and scores range between 0 and 36. Higher scores indicate greater problems with gambling. Cronbach’s alpha in the present study was excellent (.91).

Most of the 12 items (see Table 1) comprise actual gambling behavior that has been associated with problem gambling in the empirical literature. Item 1 comprises frequent depositing (i.e., reloading electronic wallets during a gambling session) which has been identified as indicative of problem gambling (Challet-Bouju et al., 2020; Delfabbro et al., 2023; Hing et al., 2015; Ukhov et al., 2021). Items 2 and 3 both comprise chasing losses which is a common item in problem gambling screens (e.g., Orford et al., 2010; American Psychiatric Association, 2013) and has been empirically tested as being associated with problem gambling using account-based tracking data (Auer & Griffiths, 2022a; Catania & Griffiths, 2022). Item 4 refers to gambling intensity (gambling for more than 4 hours a day) and is similar to items in other problem gambling screens such as the DSM-5 criteria (American Psychiatric Association, 2013) and Consumption Screen for Problem Gambling (Rockloff, 2012). Item 5 (playing a variety of stakes has been identified as a potential risk factor for problem gambling and can indicate poor planning (Delfabbro et al., 2012; Griffiths & Whitty, 2010). Item 6 (wagering on more than five game types) is another indicator of gambling intensity (Brosowski et al., 2021) and has been associated with problem gambling in large-scale representative studies (Wardle et al., 2011a). Binde et al. (2017) also found that problem gamblers regularly participate in multiple forms of gambling. Item 7 (re-gambling winnings) is indicative of impulsivity and loss of control which are important aspects of problem gambling (Ioannidis et al., 2019). Item 8 (using multiple sources of payment) is indicative of financial problems and a recent study using account-based tracking data found that the number of registered credit cards was associated with problem gambling (Catania & Griffiths, 2022). Items 9 and 10 (aggression towards other players and making complaints to customer service about losses) have both been identified as behaviors associated with problem gambling (Catania & Griffiths (2022)). Items 11 and 12 (hitting voluntary time and money limits when gambling) are indicative of losing control and have been associated with problem gambling (Auer & Griffiths, 2022b).

### Statistical Analyses

Descriptive analysis comprised the computation of averages, percentages and correlations of demographic information as well as the answers to the nine PGSI items and the 12 OPGBI items. The construct validity of the OPGBI was tested utilizing exploratory factor analysis (EFA) with varimax rotation. Goodness of fit was evaluated via the root mean square error of approximation (RMSEA), comparative fit index (CFI), and Tucker-Lewis Index (TLI). For a good fit, values should be <0.05, >0.95, >0.95, respectively (Hu & Bentler, 1999). Values greater than 0.9 for CFI and TLI are acceptable. The R program (R Core Team, 2013) was used with the ‘lavaan-package’ (Rosseel, 2012). The OPGBI’s construct validity was evaluated using RSMEA, CFI and TLI. Additionally, several machine learning algorithms were applied to predict the four PGSI categories (no-risk, low-risk, medium-risk, high-risk) from the computed OPGBI factor scores. The algorithms used were Linear Discriminant Analysis (LDA) (Riffenburgh & Clunies-Ross, 1960), Recursive Partitioning (Rpart) (Therneau & Atkinson, 1997), Random Forest (RF) (Liaw & Wiener, 2002), Gradient Boost Machine Learning (GMBL) (Friedman, 2001), and Support Vector Machines (SVM) (Hearst et al., 1998). A 10-fold cross-validation was chosen to train the models (Kuhn, 2015) and the accuracy and kappa were used to evaluate the models. Accuracy simply refers to the number of players who were correctly classified into one of the four PGSI categories. The kappa statistic is a measure of concordance for categorical data that measures agreement relative to what would be expected by chance. Values of 1 indicate perfect agreement, while a value of zero would indicate a lack of agreement (Kuhn, 2015).

### Data Cleaning

Between February 20 and February 28, 10,425 participants completed the online survey. As the application was accessible to anybody via the internet, only responses which were collected from the online site of the Croatian lotteries were filtered. A response had to contain the string www.lutrija.hr in the referrer. The referrer indicates the origin of the participant on the world wide web just before the survey’s link was clicked. Out of the 10,425 responses 10,086 originated from the Croatian lotteries site. Players were also asked to enter their age. Participants younger then 18 years were removed from the dataset (*n* = 86) because Croatian lotteries only allows people aged 18 years or older to gamble and the ethics committee only approved data collection from adults. The data cleaning reduced the dataset to 10,000 records.

## Results

### Descriptive Analysis

Table 1 reports the frequency of each answer category for the 12 gambling behavior items and the nine PGSI items. The category ‘almost always’ was chosen most frequently for the two items on the OPBGI: *“Do you reload your wallet during an online gambling session?”* (*n* = 708) and *“Do you re-gamble your online winnings straight after you have won*?” (*n* = 715). ‘Almost always’ was least frequently chosen for two items on the OPGBI: *“Do you act aggressively in online gambling chat rooms?”* (*n* = 42) and *“Do you contact customer services to complain about your online gambling losses*?” (*n* = 40). On the PGSI, ‘almost always’ was most frequently chosen for the item *“Have you felt guilty about the way you gamble or what happens when you gamble?”* (*n* = 264) and least frequently for the item *“Have you borrowed money or sold anything to get money to gamble?”* (*n* = 97).

In relation to the type of online and/or offline gambling engaged in over the past month, the participants reported sports betting (50%), horserace betting (1.65%), betting on other events (4%), slot machines (19.9%), Lotto (72%), scratchcards (17.3%), roulette (4.6%), blackjack (1.85%), bingo (1.72%), poker (3.55%), and other forms of gambling (4.6%). On average, participants had engaged in two types of gambling in the past month (SD = 1.2). Participants were categorized into four types of gambler based on their PGSI score was classified into four categories: non-problem gamblers (53% scoring 0), low-risk gamblers (22.4% scoring 1–2), medium-risk gamblers (16.8% scoring 3–7), and problem gamblers (7.1% scoring 8 and above).

### Construct Validity of the OPGBI – Exploratory Factor Analysis

In order to test the construct validity of the OPGBI, an EFA was conducted. Kaiser-Meyer-Olkin (KMO) and Bartlett’s test indicated that the KMO measure of sampling adequacy was higher than .70 and Bartlett’s test of sphericity was significant (Kline, 2014), indicating a good structure (.87; *p* < .001). In order to find the number of latent factors, a scree test (i.e., scree plot) was performed. It is recommended that factors should be retained if they have an eigenvalue >1 (Kaiser, 1960; Yong & Pearce, 2013). Parallel analysis (Horn, 1965) compares the generated data’s eigenvalues to those generated from simulated Monte-Carlo data. The optimal coordinates and the acceleration factor are non-graphical approaches in which the number of factors can be determined (Raiche et al., 2006). Both these methods can locate the point in the scree plot where there are the most abrupt changes in the slope of the curve. Supplementary Figure 1 indicates the 12 items comprise three factors. Therefore, these factors were further evaluated by carrying an EFA.

Table 2 reports the factor loadings for the 12 items and three factors of the OPGBI. The column ‘H^2^’ is the communality (explained variance) for each item. Factor loadings >0.4 are displayed. The three factors explained 46% of the variance of the OPGBI and Factor 1 summarizes all gambling behavioral specific questions had the highest eigenvalue (2.9). Item 12 (*“Do you hit your [or the website’s] money spending limits [if you have any]?”)* had the highest communality (1%). Item 8 (*“Do you use different debit or credit cards to load up your wallet during an online gambling session?”*) did not load clearly on one of the three factors as none of the loadings were greater than 0.4.

**Table 2.** Factor Loadings for the Three Factor EFA Solution With Varimax Rotation on the OPGBI

Number	Item	Gambling Behavior	Limits	Communication	H^2^
1	Do you reload your wallet during an online gambling session?	0.52	-	-	0.29
2	Do you increase your stakes after losing in an online gambling session?	0.69	-	-	0.51
3	Do you increase your stakes the following day after you have lost in an online gambling session?	0.66	-	-	0.52
4	Do you gamble online for longer than 4 hours a day?	0.55	-	-	0.42
5	Do you gamble online with a variety of different stakes?	0.64	-	-	0.44
6	Do you play more than five types of online gambling games in a month?	0.55	-	-	0.36
7	Do you re-gamble your online winnings straight after you have won?	0.57	-	-	0.36
8	Do you use different debit or credit cards to load up your wallet during an online gambling session?	-	-	-	0.19
9	Do you act aggressively in online gambling chat rooms?	-	-	0.70	0.52
10	Do you contact customer services to complain about your online gambling losses?	-	-	0.62	0.42
11	Do you hit your (or the website’s) money spending limits (if you have any)?	-	0.97	-	0.1
12	Do you hit your (or the website’s) time spending limits (if you have any)?	-	0.61	-	0.51
	Eigenvalue	2.84	1.48	1.2	5.52
	Explained percentage	22%	12%	10%	46%

The three-factor solution’s goodness of fit statistics were: RMSEA: 0.077 (0.074–0.08); TLI: 0.891; chi-square: 1,985 (*p* < 0.001, df = 33). The *p*-value for the chi-square test (<.05) was expected to be non-significant. Nevertheless, chi-square results are sensitive to large sample sizes (*n* > 200), sometimes producing false positives, in which case it is recommended to weigh the indicators of the rest of the fit exams before discarding the proposed model (Hair et al., 2010). Cronbach’s alpha reliability coefficient was 0.82 (0.82–0.83). Regardless of sample size, Stevens (1992) recommends using a cut-off of 0.4 for factor loadings. Following thes recommendations, the following interpretations can be derived based on the factor loadings:

- F1 (*Gambling Behavior*): This factor loaded on seven out of the 12 items and summarizes all the gambling behavior related questions.
- F2 (*Limits*): This factor loaded on the two items referring to limit-setting.
- F3 (*Communication*): This factor loaded on two items referring to communication with gambling operators.

### External Validity

In order to compute the OPGBI score the 12 respective item values were summed up for each participant. The sum of the 12 OPGBI items ranges between 0 and 36. The PGSI score ranges from 0 to 27. The correlation between OPGBI and PGSI was highly significant (*r* = 0.68). Next, the three factor scores were computed for each player and correlated with the PGSI score. Table 3 reports a linear multiple regression with the three factor scores as explanatory variables. The *R*^2^ was 0.517 (F = 3,567, df = 3, *p* < 0.001) which means that the three-factor model explained 51.7% of the variance of the PGSI score. The bivariate correlations between the three factors and the PGSI score were 0.59, 0.20 and 0.25. Table 4 reports the results of a multiple regression model which includes all available variables and the PGSI score as dependent variable. Except for the gender category ‘other’ and the three types of gambling (blackjack, sports betting, and poker) and ‘other’ forms of gambling, all variables were significantly correlated with the PGSI score. The *R*^2^ was 0.54 (F = 700, df = 15, *p* < 0.001). An ANOVA comparison of the two models demonstrated that the multiple regression with the additional variables was significantly better than the model which only contained the three factors (F = 41.85, df = 12, *p* < 0.001).

**Table 3.** Linear Model With the Three Factors as Explanatory Variables and the PGSI Score as Dependent Variable

Explanatory Variable	Estimate	Std.Error	*t*	*p*
Intercept	1.94	0.026	75	<0.001
Factor 1: Gambling behavior	2.30	0.03	78	<0.001
Factor 2: Limit-setting	0.74	0.026	29	<0.001
Factor 3: Communication	1.54	0.033	47	<0.001

**Table 4.** Linear Model With all Variables as Explanatory Variables and the PGSI Score as Dependent Variable

Explanatory Variable	Estimate	Std. Error	*t*-value	*p*-value
Intercept	2.2199	0.0671	33.074	<0.001
Factor 1: Gambling behavior	1.9674	0.0329	59.882	<0.001
Factor 2: Limit-setting	0.8209	0.0281	29.165	<0.001
Factor communication	1.3828	0.0330	41.881	<0.001
Blackjack	−0.3890	0.2490	−1.563	0.118
Sports betting	−0.1457	0.1519	−0.959	0.338
Poker	0.2674	0.2070	1.292	0.196
Lotto	−0.9650	0.1515	−6.370	<0.001
Other forms of gambling	−0.1784	0.1903	−0.937	0.349
Scratchcards	−0.4149	0.1555	−2.669	0.007
Bingo	−0.5329	0.1558	−3.421	<0.001
Slot machines	0.5095	0.1535	3.320	<0.001
Roulette	0.5279	0.1929	2.737	0.006
Horserace betting	1.0382	0.2675	3.882	<0.001
Age	−0.0313	0.0019	−6.999	<0.001
Female	−0.2006	0.0622	−3.227	0.001
Gender: Other	0.1442	0.2985	0.470	0.638
Number of game types	0.2786	0.1359	2.050	0.04

Five machine learning algorithms, LDA, Rpart, SVM, RF, and GMBL were used to predict the four PGSI categories based on the three OPGBI scores. Supplementary Tables 1 and 2 report the distribution of the accuracy and kappa statistics for each of the machine learning models. Each machine learning model was run through several iterations which resulted in a distribution of goodness of fit statistics (accuracy and kappa). In every iteration, a hold-out sample was used to compute the goodness of fit. This procedure protects the results from reflecting overfitted prediction accuracies. SVM and GBML had the highest median and mean accuracy and kappa and were therefore selected. Supplementary Tables 3 and 4 report sensitivity, specificity, and accuracy for SVM and GBML.

Sensitivity refers to the true positive rate which is the probability of a participant in a specific category being predicted as belonging to that category. A total of 73% and 71% of high-risk participants were correctly classified by the two algorithms. A total of 49% and 45% of moderate-risk participants were correctly classified by the two algorithms. A total of 43% and 38% of low-risk participants were correctly classified by the two algorithms. Finally, a total of 69% of no-risk participants were correctly classified by both algorithms. Specificity refers to the true negative rate which is the probability of a participant who is not in a specific category being predicted not to belong to that category. A total of 97% and 95% of participants who were not high-risk were also predicted not to be high-risk. A total of 89% of participants who were not moderate-risk were also predicted not to be moderate-risk by both algorithms. A total of 79% of participants who were not low-risk were also predicted not to be low-risk by both algorithms. Finally, 85% and 84% of participants who were not low-risk were also predicted not to be low-risk. Supplementary Tables 3 and 4 also report the overall classification accuracy for each of the four PGSI categories. A total of 85% and 84% of high-risk participants were correctly classified, 69% and 67% of moderate-risk participants were correctly classified, 61% and 59% of low-risk participants were correctly classified, and 77% and 76% of no-risk participants were correctly classified.

Two OPGBI items *“Do you increase your stakes after losing in an online gambling session?”* and *“Do you increase your stakes after losing in an online gambling session?”* assess chasing losses. Item 3 *“Have you gone back to try to win to back the money you’d lost?”* of the PGSI also assesses chasing losses. The two OPGBI items correlations with the PGSI item were 0.49 and 0.53, respectively (Supplementary Table 5).

### PGSI Construct Validity

The PGSI’s construct validity was also tested with a confirmatory factor analysis (CFA) on the 10,000 Croatian participants. The goodness of fit statistics for a one-factor solution were as follows: RMSEA: 0.10 (0.103–0.106); TLI: 0.926; CFI: 0.944; chi-square: 2,875 (*p* < 0.001, df = 27). Supplementary Figure 2 displays a scree-test which indicates that the nine PGSI items appear to be explained by one underlying latent factor. The one-factor solution explained 56% of the variance and Cronbach’s alpha reliability coefficient was 0.91 (0.91–0.92).

## Discussion

The present study developed a new screen – the OPGBI – and examined its psychometric properties. EFA showed that the 12 items produced three latent factors. The first factor describes the majority of the items related to gambling behavior, the second factor captures two items assessing limit-setting, and the third factor describes communication with operators. One item (*“Do you use different debit or credit cards to load up your wallet during an online gambling session?”*) could not be assigned to any factor and their communalities were also very low.

The OPGBI score was significantly correlated with the PGSI score (*r* = 0.68). The three OPGBI factor scores were also significantly correlated with the PGSI score (*r* = 0.72). This slightly higher correlation means that the OPGBI factor scores have a higher explanatory power of the PGSI score than the OPGBI score itself. This is particularly interesting as the three latent factors can never contain as much information as the 12 underlying items. Factor analysis is a dimension reduction procedure and obviously the “noise” which was contained in the 12 items and was removed by the factor analysis lowered the explanatory power of the OPGBI score with respect to the PGSI score. Given that the OPGBI items are not assessing any psychological aspects of problem gambling it is encouraging that they explained approximately 52% of the nine PGSI questions. It also needs to be taken into account that none of the OPGBI items ask about psychological or emotional aspects of gambling nor do they assess consequences of problem gambling. The psychometric analyses suggest that actual gambling behavior (as assessed using the OPGBI) is predictive of problem gambling and therefore supports assumptions made by previous research (e.g., Catania & Griffiths, 2022; Griffiths, 2010; Delfabbro et al., 2012; Ukhov et al., 2021). A similar study which investigated a new gambling screen based mostly on questions related to emotions found a slightly higher correlation with the PGSI (*r* = 0.81) than the correlation found with the OPGBI (Jonsson et al., 2017). This small difference is probably due to the fact that the OPGBI items ask exclusively about actual gambling behavior rather than the consequences of it.

Apart from correlating the OPGBI factors with the overall PGSI score, several machine learning algorithms were used to predict the four PGSI categories based on the three OPGBI factors. SVM and GBML seemed to predict the PGSI categories most accurately. The high-risk and no-risk categorieshad the highest prediction accuracy, whereas moderate-risk and low-risk could not be predicted as accurately. Moreover, 73% and 71% of players who were actually high-risk were also predicted to be high risk by the SVM and GBML, and 97% and 96% of players who were not high-risk were also predicted to belong to another category by SVM and GBML. Therefore, the high-risk category had the highest sensitivity (true positive) and highest specificity (true negative) compared to the other three PGSI categories. There seemed to be a non-linear relationship between prediction accuracy and risk category. High and low risk can be predicted more accurately than moderate and low risk. It could be due to the fact that participants in the very extremes are more specific with respect to their answers compared to participants in the middle.

The study also supports the findings by Haefeli et al. (2011, 2015) who found that content of communications with online gambling operators were predictive of problem gambling. The present study also showed a correlation between problem gambling and game-types. Gambling with slot machines, roulette and horserace betting were positively correlated with problem gambling (as assessed by the PGSI score). Slot machine gambling (reported by 20% of the participants in the past month) has been associated with increased problem gambling in other studies (e.g., Binde, 2011; Dowling et al., 2005; Griffiths, 1994; Holtgraves, 2009; MacLaren, 2016; Wardle et al. 2011b; Williams et al., 2012). One reason for this elevated association with problem gambling compared to other gambling activities is the high event frequency and speed of play (Harris & Griffiths, 2018).

Very few players reported having gambled on roulette or horserace betting in the past month. Lotto games were negatively correlated with problem gambling which is also in support of previous research (e.g., Griffiths & Wood, 2001; Haw, 2008; Linnet et al., 2010). The number of reported game-types was also significant in the multiple regression model predicting the PGSI score. In their player tracking study of 4,056 real-world online gamblers, Braverman et al. (2011) also concluded that increased number of game-types was positively correlated with problem gambling.

Item 3 of the PGSI *“Have you gone back to try to win to back the money you’d lost?”* assesses chasing losses and it might be assumed that there would be a high correlation with the two OPGBI items *“Do you increase your stakes after losing in an online gambling session?”* and *“Do you increase your stakes after losing in an online gambling session?”.* However, a little less than 75% of the variance is unexplained. Whereas the OPGBI chasing losses items are very specific, the PGSI chasing losses item is very general. There is no commonly accepted definition of chasing losses and the results in the present study show that the response depends on the specificity and content of the item. The present study also tested the PGSI’s construct validity and confirmed the results of previous studies demonstrating that the PGSI is unidimensional (Lopez-Gonzalez et al., 2018; So et al., 2019).

### Limitations

The present study is not without limitations. The study was conducted with online players from one gambling operator. The gambling operator runs online lottery as well as offering casino games. Consequently, such players may be different from purely casino-focused operators. Future studies concerning the OPGBI should be applied on samples from different operators and different geographical regions. Operators also vary with respect to responsible gambling tools such as limit setting, mandatory play breaks, and self-exclusions which might influence a player’s understanding of some of the OPGBI items. The study was also conducted at one specific period of time and although the authors were not aware of any external significant events, the study should be replicated in order to exclude significant time-related events.

## Conclusion

The fact that purely behavioral based aspects of gambling are predictive of psychological aspects and consequently problematic gambling could be of great advantage to gambling operators. Gambling behavioral is readily available to online operators and account-based land-based operators and for that reason they could easily identify the 12 behaviors in the OPGBI. Further research should compare of self-reported gambling with actual gambling data. This way actual gambling behavior could be compared to the OPGBI responses and PGSI categories could be predicted. To the authors’ knowledge this has never been done before. To date, studies have used self-exclusion as proxy measures for problematic gambling (Haeusler, 2016; Percy et al., 2016; Dragicevic et al., 2015). However, self-exclusion is not entirely the same as problematic gambling (Auer & Griffiths, 2016; Catania & Griffiths, 2021; Hopfgartner et al., 2023). The scale will also be of use to health practitioners involved in therapeutic interventions of individuals with gambling problems (particularly psychologists, psychiatrists, addiction specialists, and treatment providers) who can use the scale alongside other diagnostic screens (e.g., DSM-5, PGSI). Unlike the most used problem gambling diagnostic screens, the OPGBI focuses on actual gambling behavior and can be a useful adjunct to help in the targeting therapeutic goal-setting to reduce very specific types of online gambling behaviors. Future studies should also try to develop an offline equivalent of the OPGBI because some of the items in the OPGBI are very specific to online gambling (e.g., *“Do you reload your wallet during an online gambling session?”*) and offline equivalents would need to be developed (e.g., *“Do you go to the ATM machine during a gambling session to get money to continue gambling?”*).

## Supplemental Material

Supplemental Material - Development of the Online Problem Gaming Behavior Index: A New Scale Based on Actual Problem Gambling Behavior Rather Than the Consequences of it Click here for additional data file. Supplemental Material for Development of the Online Problem Gaming Behavior Index: A New Scale Based on Actual Problem Gambling Behavior Rather Than the Consequences of it by Michael Auer, Neven Ricijas, Valentina Kranzelic, and Mark D. Griffiths in Evaluation & the Health Professions
