# Supplementary material for: Development of the Online Problem Gaming Behavior Index: A New Scale Based on Actual Problem Gambling Behavior Rather Than the Consequences of it
Source: Eval Health Prof. 2023 May 27;47(1):81–92. doi: 10.1177/01632787231179460 (PMC10858630; doi:10.1177/01632787231179460)
Supplement: Supplemental Material - Development of the Online Problem Gaming Behavior Index: A New Scale Based on Actual Problem Gambling Behavior Rather Than the Consequences of it [file sj-pdf-1-ehp-10.1177_01632787231179460.pdf]

Supplementary Materials

Supplementary Figure 1

***Scree plot of the OPGBI items displaying the four goodness of fit criteria for determining the number of factors*[1]**

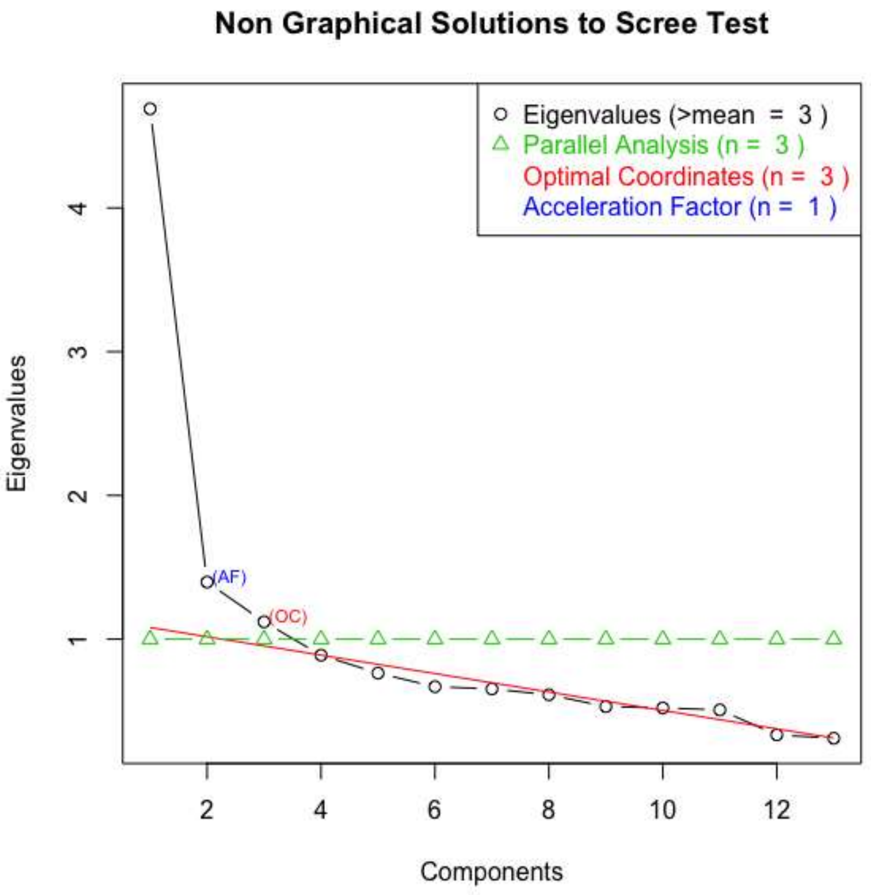

[1] Line with 'dots' displays eigenvalues. Line with 'triangles' represents parallel analysis. Cut-off for acceleration factor and optimal coordinate are indicated in the Figure.

Supplementary Figure 2

Scree plot of the nine PGSI items displaying the four goodness of fit criteria for determining the number of factors. Line with 'dots' displays eigenvalues. Line with 'triangles' represents parallel analysis. Cut-off for acceleration factor and optimal coordinate are indicated in the Figure.

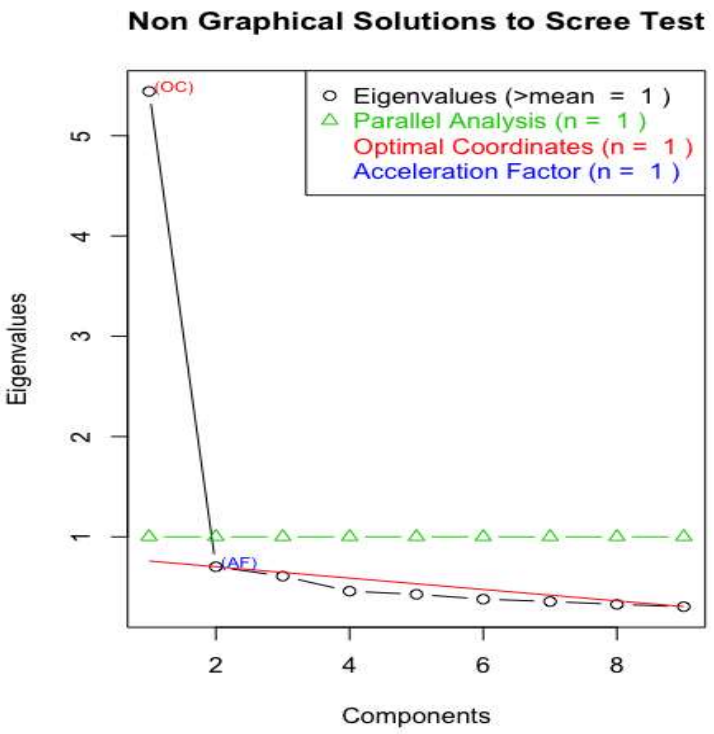

Supplementary Table 1

Distribution of the correctly classified participants' PGSI category for the five prediction models[2]

	Min.	Q1	Median	Mean	Q3	Max
LDA	0.596	0.600	0.607	0.607	0.615	0.620
RPART	0.568	0.580	0.592	0.591	0.601	0.614
SVM	0.599	0.605	0.615	0.613	0.620	0.626
RF	0.571	0.584	0.592	0.591	0.599	0.606
GBML	0.598	0.601	0.618	0.612	0.622	0.624

Supplementary Table 2

[2] Each model consists of several training iterations and computes the accuracy each time

Distribution of the kappa metric for the five prediction models[3]

	Min.	Q1	Median	Mean	Q3	Max
LDA	0.250	0.256	0.265	0.268	0.281	0.288
RPART	0.227	0.262	0.278	0.277	0.299	0.312
SVM	0.297	0.303	0.323	0.318	0.328	0.343
RF	0.275	0.289	0.305	0.300	0.308	0.319
GBML	0.284	0.303	0.326	0.317	0.333	0.335

Supplementary Table 3

Sensitivity, specificity and accuracy for the SVM algorithm

	High	Moderate	Low	No
Sensitivity	0.73	0.49	0.43	0.69
Specificity	0.97	0.89	0.79	0.85
Balanced Accuracy	0.85	0.69	0.61	0.77

Supplementary Table 4

Sensitivity, specificity and accuracy for the GBML algorithm

	High	Moderate	Low	No
Sensitivity	0.73	0.49	0.43	0.69
Specificity	0.97	0.89	0.79	0.85
Balanced Accuracy	0.85	0.69	0.61	0.77

Appendix 1

English OPGBI survey items

[3] The kappa metric measures prediction accuracy but it also takes into account the distribution of the category. The four PGSI categories are not equally large. 1 indicates perfect fit and 0 indicates a bad fit

Item Number	Item
1	Do you reload your wallet during an online gambling session?
2	Do you increase your stakes after losing in an online gambling session?
3	Do you increase your stakes the following day after you have lost in an online gambling session?
4	Do you gamble online for longer than four hours a day?
5	Do you gamble online with a variety of different stakes?
6	Do you play more than five types of online gambling games in a month?
7	Do you re-gamble your online winnings straight after you have won?
8	Do you use different debit or credit cards to load up your wallet during an online gambling session?
9	Do you act aggressively in online gambling chat rooms?
10	Do you contact customer services to complain about your online gambling losses?
11	Do you hit your (or the website's) money spending limits (if you have any)?
12	Do you hit your (or the website's) time spending limits (if you have any)?

Appendix 2

Croatian OPGBI survey items

Item Number	Item
1	Nadopunjujete li svoj račun tijekom online kockanja?
2	Povećavate li svoje uloge nakon gubitka u online kockanju?
3	Povećavate li svoje uloge sljedećeg dana nakon što ste izgubili u online kockanju?
4	Kockate li online više od četiri sata dnevno?
5	Kockate li online s više različitih ulaganja?
6	Igrate li više od pet vrsta online kockarskih igara u mjesecu?
7	Kockate li odmah s novcem osvojenim online kockanjem?
8	Koristite li različite kreditne ili debitne kartice kako biste napunili svoj račun tijekom online kockanja?
9	Ponašate li se agresivno u online komunikaciji s drugim igračima tijekom online kockanja, ukoliko ta opcija postoji?
10	Kontaktirate li službu za korisnike kako biste se žalili na gubitke prilikom online kockanja?
11	Dosegnete li vlastiti limit potrošnje novca ako ste ga postavili (ili onaj postavljen na mrežnoj stranici)?
12	Dosegnete li vlastiti limit vremena kockanja (ili onaj postavljen na mrežnoj stranici) ukoliko ta opcija postoji?

Supplementary Table 5

Correlation Matrix including PGSI Score, OPGBI Score and the three latent factors of the 12 behavioral questions

	1	2	3	4	5	6	7	8	9	10	11	12	1	2	3	4	5	6	7	8	9	age	female	other	num_gametypes	PGSI	PGBI	F1	F2	F3
1	1.00	0.36	0.32	0.30	0.34	0.27	0.35	0.32	0.10	0.10	0.22	0.21	0.25	0.24	0.30	0.21	0.27	0.21	0.22	0.23	0.27	-0.07	-0.01	-0.01	0.11	0.32	0.61	0.59	0.12	0.00
2	0.36	1.00	0.66	0.42	0.45	0.38	0.37	0.27	0.23	0.19	0.27	0.30	0.46	0.44	0.49	0.37	0.42	0.38	0.36	0.39	0.42	-0.16	-0.05	0.02	0.23	0.54	0.68	0.78	0.11	0.21
3	0.32	0.66	1.00	0.48	0.44	0.39	0.36	0.28	0.29	0.26	0.30	0.34	0.49	0.48	0.53	0.42	0.43	0.40	0.39	0.43	0.41	-0.14	-0.03	0.03	0.20	0.57	0.69	0.75	0.13	0.33
4	0.30	0.42	0.48	1.00	0.41	0.44	0.32	0.24	0.31	0.29	0.28	0.33	0.43	0.40	0.45	0.42	0.40	0.36	0.36	0.41	0.37	-0.06	0.02	0.02	0.15	0.51	0.63	0.62	0.13	0.40
5	0.34	0.45	0.44	0.41	1.00	0.45	0.45	0.25	0.21	0.20	0.29	0.28	0.36	0.36	0.42	0.31	0.36	0.30	0.33	0.31	0.37	-0.13	-0.06	0.02	0.28	0.45	0.68	0.72	0.14	0.16
6	0.27	0.38	0.39	0.44	0.45	1.00	0.36	0.23	0.23	0.22	0.24	0.28	0.35	0.35	0.39	0.30	0.34	0.28	0.29	0.31	0.31	-0.09	0.03	0.03	0.29	0.42	0.63	0.62	0.11	0.26
7	0.35	0.37	0.36	0.32	0.45	0.36	1.00	0.31	0.15	0.14	0.29	0.27	0.30	0.33	0.39	0.25	0.30	0.26	0.26	0.26	0.33	-0.07	-0.02	-0.01	0.24	0.39	0.66	0.64	0.17	0.06
8	0.32	0.27	0.28	0.24	0.25	0.23	0.31	1.00	0.18	0.16	0.23	0.22	0.29	0.27	0.29	0.26	0.26	0.24	0.23	0.26	0.27	-0.04	0.00	-0.01	0.15	0.34	0.56	0.44	0.15	0.14
9	0.10	0.23	0.29	0.31	0.21	0.23	0.15	0.18	1.00	0.48	0.24	0.29	0.36	0.34	0.29	0.44	0.28	0.36	0.32	0.37	0.23	-0.08	-0.04	0.07	0.15	0.41	0.41	0.17	0.13	0.88
10	0.10	0.19	0.26	0.29	0.20	0.22	0.14	0.16	0.48	1.00	0.23	0.25	0.30	0.27	0.25	0.34	0.24	0.29	0.25	0.29	0.20	-0.06	-0.03	0.06	0.12	0.34	0.38	0.16	0.12	0.78
11	0.22	0.27	0.30	0.28	0.29	0.24	0.29	0.23	0.24	0.23	1.00	0.68	0.31	0.30	0.33	0.28	0.29	0.27	0.26	0.28	0.28	0.00	-0.01	0.01	0.14	0.37	0.59	0.24	0.98	0.16
12	0.21	0.30	0.34	0.33	0.28	0.28	0.27	0.22	0.29	0.25	0.68	1.00	0.37	0.35	0.36	0.34	0.32	0.32	0.29	0.33	0.31	-0.03	-0.01	0.01	0.15	0.43	0.60	0.32	0.61	0.30
1	0.25	0.46	0.49	0.43	0.36	0.35	0.30	0.29	0.36	0.30	0.31	0.37	1.00	0.60	0.59	0.62	0.53	0.55	0.48	0.61	0.53	-0.19	-0.03	0.03	0.19	0.78	0.57	0.50	0.19	0.37
2	0.24	0.44	0.48	0.40	0.36	0.35	0.33	0.27	0.34	0.27	0.30	0.35	0.60	1.00	0.58	0.54	0.50	0.52	0.46	0.51	0.49	-0.16	-0.03	0.04	0.23	0.74	0.55	0.49	0.19	0.34
3	0.30	0.49	0.53	0.45	0.42	0.39	0.39	0.29	0.29	0.25	0.33	0.36	0.59	0.58	1.00	0.52	0.54	0.51	0.49	0.52	0.56	-0.17	-0.01	0.02	0.23	0.77	0.62	0.58	0.20	0.29
4	0.21	0.37	0.42	0.42	0.31	0.30	0.25	0.26	0.44	0.34	0.28	0.34	0.62	0.54	0.52	1.00	0.51	0.57	0.54	0.67	0.49	-0.11	-0.04	0.04	0.18	0.76	0.51	0.41	0.17	0.45
5	0.27	0.42	0.43	0.40	0.36	0.34	0.30	0.26	0.28	0.24	0.29	0.32	0.53	0.50	0.54	0.51	1.00	0.61	0.60	0.59	0.66	-0.10	-0.08	0.04	0.18	0.80	0.53	0.48	0.18	0.28
6	0.21	0.38	0.40	0.36	0.30	0.28	0.26	0.24	0.36	0.29	0.27	0.32	0.55	0.52	0.51	0.57	0.61	1.00	0.58	0.63	0.58	-0.10	-0.07	0.05	0.16	0.79	0.48	0.40	0.17	0.36
7	0.22	0.36	0.39	0.36	0.33	0.29	0.26	0.23	0.32	0.25	0.26	0.29	0.48	0.46	0.49	0.54	0.60	0.58	1.00	0.58	0.54	-0.10	-0.09	0.04	0.19	0.75	0.47	0.41	0.16	0.32
8	0.23	0.39	0.43	0.41	0.31	0.31	0.26	0.26	0.37	0.29	0.28	0.33	0.61	0.51	0.52	0.67	0.59	0.63	0.58	1.00	0.58	-0.10	-0.02	0.05	0.16	0.80	0.51	0.43	0.17	0.38
9	0.27	0.42	0.41	0.37	0.37	0.31	0.33	0.27	0.23	0.20	0.28	0.31	0.53	0.49	0.56	0.49	0.66	0.58	0.54	0.58	1.00	-0.14	-0.06	0.01	0.20	0.79	0.52	0.48	0.18	0.22
age	-0.07	-0.16	-0.14	-0.06	-0.13	-0.09	-0.07	-0.04	-0.08	-0.06	0.00	-0.03	-0.19	-0.16	-0.17	-0.11	-0.10	-0.10	-0.10	-0.10	-0.14	1.00	-0.08	0.05	-0.09	-0.17	-0.12	-0.16	0.04	-0.08
female	-0.01	-0.05	-0.03	0.02	-0.06	0.03	-0.02	0.00	-0.04	-0.03	-0.01	-0.01	-0.03	-0.03	-0.01	-0.04	-0.08	-0.07	-0.09	-0.02	-0.06	-0.08	1.00	-0.05	-0.04	-0.06	-0.03	-0.03	0.00	-0.03
other	-0.01	0.02	0.03	0.02	0.02	0.03	-0.01	-0.01	0.07	0.06	0.01	0.01	0.03	0.04	0.02	0.04	0.04	0.05	0.04	0.05	0.01	0.05	-0.05	1.00	0.05	0.04	0.02	0.01	0.00	0.08
num_gametypes	0.11	0.23	0.20	0.15	0.28	0.29	0.24	0.15	0.15	0.12	0.14	0.15	0.19	0.23	0.23	0.18	0.18	0.16	0.19	0.16	0.20	-0.09	-0.04	0.05	1.00	0.25	0.31	0.29	0.08	0.13
PGSI	0.32	0.54	0.57	0.51	0.45	0.42	0.39	0.34	0.41	0.34	0.37	0.43	0.78	0.74	0.77	0.76	0.80	0.79	0.75	0.80	0.79	-0.17	-0.06	0.04	0.25	1.00	0.68	0.61	0.23	0.42
PGBI	0.61	0.68	0.69	0.63	0.68	0.63	0.66	0.56	0.41	0.38	0.59	0.60	0.57	0.55	0.62	0.51	0.53	0.48	0.47	0.51	0.52	-0.12	-0.03	0.02	0.31	0.68	1.00	0.88	0.41	0.37
F1	0.59	0.78	0.75	0.62	0.72	0.62	0.64	0.44	0.17	0.16	0.24	0.32	0.50	0.49	0.58	0.41	0.48	0.40	0.41	0.43	0.48	-0.16	-0.03	0.01	0.29	0.61	0.88	1.00	0.04	0.15
F2	0.12	0.11	0.13	0.13	0.14	0.11	0.17	0.15	0.13	0.12	0.98	0.61	0.19	0.19	0.20	0.17	0.18	0.17	0.16	0.17	0.18	0.04	0.00	0.00	0.08	0.23	0.41	0.04	1.00	0.03
F3	0.00	0.21	0.33	0.40	0.16	0.26	0.06	0.14	0.88	0.78	0.16	0.30	0.37	0.34	0.29	0.45	0.28	0.36	0.32	0.38	0.22	-0.08	-0.03	0.08	0.13	0.42	0.37	0.15	0.03	1.00
